# Knowledge, attitudes, and current practices toward lung cancer palliative care management in China: a national survey

**DOI:** 10.3389/fonc.2024.1382496

**Published:** 2024-05-15

**Authors:** Mengting Chen, Suocheng Hui, Yalan Huang, Huiqing Yu, Hong Yang, Liejun Yang, Ling Tian, Sixiong Wang

**Affiliations:** ^1^ Department of Clinical Nutrition, Chongqing University Cancer Hospital, School of Medicine, Chongqing University, Chongqing, China; ^2^ Department of Clinical Nutrition, The People’s Hospital of Chongqing Liang Jiang New Area, Chongqing, China; ^3^ Outpatient Department, Yunnan Provincial Corps Hospital of Chinese People’s Armed Police Forces, Kunming, Yunnan, China; ^4^ Department of Geriatric Oncology and Department of Palliative Care, Chongqing University Cancer Hospital, School of Medicine, Chongqing University, Chongqing, China

**Keywords:** palliative care, China, surveys and questionnaires, pain management, health personnel

## Abstract

**Scope:**

The present investigation seeks to illuminate the current state and disparities in the knowledge, attitudes, and practices (KAP) among healthcare professionals regarding the management of lung cancer palliative care (LCPC) in China, while simultaneously assessing the prevalence and context of patient-controlled analgesia (PCA) usage in the management of cancer-related pain.

**Methods:**

A total of 2093 healthcare practitioners from 706 hospitals across China completed a structured questionnaire that probed various facets of LCPC management. The questionnaire consisted of seven thematic sections, incorporating chi-square tests and Fisher’s exact probabilities to statistically assess the discrepancies in KAP among healthcare professionals across different hospital grades. Ordered data distributions among hospital grades were compared using non-parametric Kruskal-Wallis H and Mann-Whitney U tests. Multiple-choice items were subjected to multiple-response cross-tabulation analysis, while the Spearman rank-order correlation coefficient was employed to gauge potential associations among variables.

**Results:**

Around 84.2% of the respondents perceived anti-tumor therapy to be of equal importance to palliative care. Statistically significant differences (χ² = 27.402, *P* = 0.002) in satisfaction levels were observed, with participants from Tertiary hospitals demonstrating higher satisfaction compared to those from Secondary and Primary hospitals. Pain emerged as the most prevalent symptom necessitating LCPC. Major impediments to LCPC adoption included patients’ and families’ concerns about the safety of long-term palliative care-related drug use. 31.1% of the respondents cited the most frequent rationale for PCA use as cases involving patients who required systemic administration of large opioid doses or exhibited intolerable adverse reactions to opioids. The principal deterrents against the use of PCA for cancer pain management were (1): apprehension about adverse drug reactions due to overdose (2), concern about the potential for opioid addiction, and (3) the anticipated increase in patients’ economic burdens. Over the preceding 24-month period, 33.9% of the surveyed healthcare practitioners reported no engagement in either online or offline LCPC-related training initiatives.

**Conclusion:**

This study emphasizes the pressing need for comprehensive training in LCPC among Chinese health personnels, particularly focusing on the effective management of cancer pain symptoms.

## Introduction

An estimated 56.8 million individuals globally are in dire need of palliative care annually, yet only a paltry 12% receive such care (1). Cancer, representing 28.2% of all medical conditions necessitating palliative care (2), stands as the dominant disease category demanding such services. A recent report underscores that China shoulders the world’s most substantial burden of lung cancer, topping the charts in both incidence and mortality rates (3). Distressingly, close to 80% of non-small-cell lung cancer cases are diagnosed at a late stage, effectively ruling out the feasibility of optimal surgical intervention (4). As a direct consequence, the inherent limitations in the efficacy of advanced lung cancer treatments contribute to an unsatisfactory 5-year survival rate (4, 5), thereby accentuating the urgent need for palliative care services within China (6–9).

Despite the firmly entrenched status of palliative care in high-income nations, its provision in low- and middle-income countries (LMICs) persistently falls short, engendering a substantial and morally disconcerting inequality (10–12). This pronounced gap is largely attributed to the pervasive lack of specialized palliative care training among healthcare providers operating within LMICs contexts (13–16).

Acknowledging the paramount importance of health personnels’ knowledge, attitudes, and practices (KAP) in determining the quality and accessibility of LCPC services, this study endeavors to thoroughly examine these critical factors. The knowledge base of health personnels regarding palliative care principles, their prevailing attitudes toward its delivery, and their daily practices collectively exert a profound impact on patient outcomes, the efficiency of service provision, and the overarching functionality of palliative care frameworks (13). A thorough comprehension of health personnels’ KAP thus holds the potential to inform the design of targeted interventions aimed at refining clinical practice, rectifying identified challenges, and ultimately fostering the overall enhancement of LCPC service performance.

This pioneering study constitutes the inaugural inquiry into LCPC management within the Chinese context. Consequently, this nationwide descriptive cross-sectional study seeks to bridge this knowledge gap by delving into the current KAP landscape among healthcare professionals actively engaged in LCPC management within China. This inquiry promises to yield invaluable insights into the prevailing strengths, vulnerabilities, and opportunities for enhancement within the Chinese LCPC milieu, as well as the realm of patient-controlled analgesia (PCA) in cancer pain management. Such insights will serve as a compass for future capacity-building endeavors and policy deliberations aimed at ameliorating the disparities in access to high-quality palliative care services for lung cancer patients.

## Method

### Study design and setting

**Figure 1:**
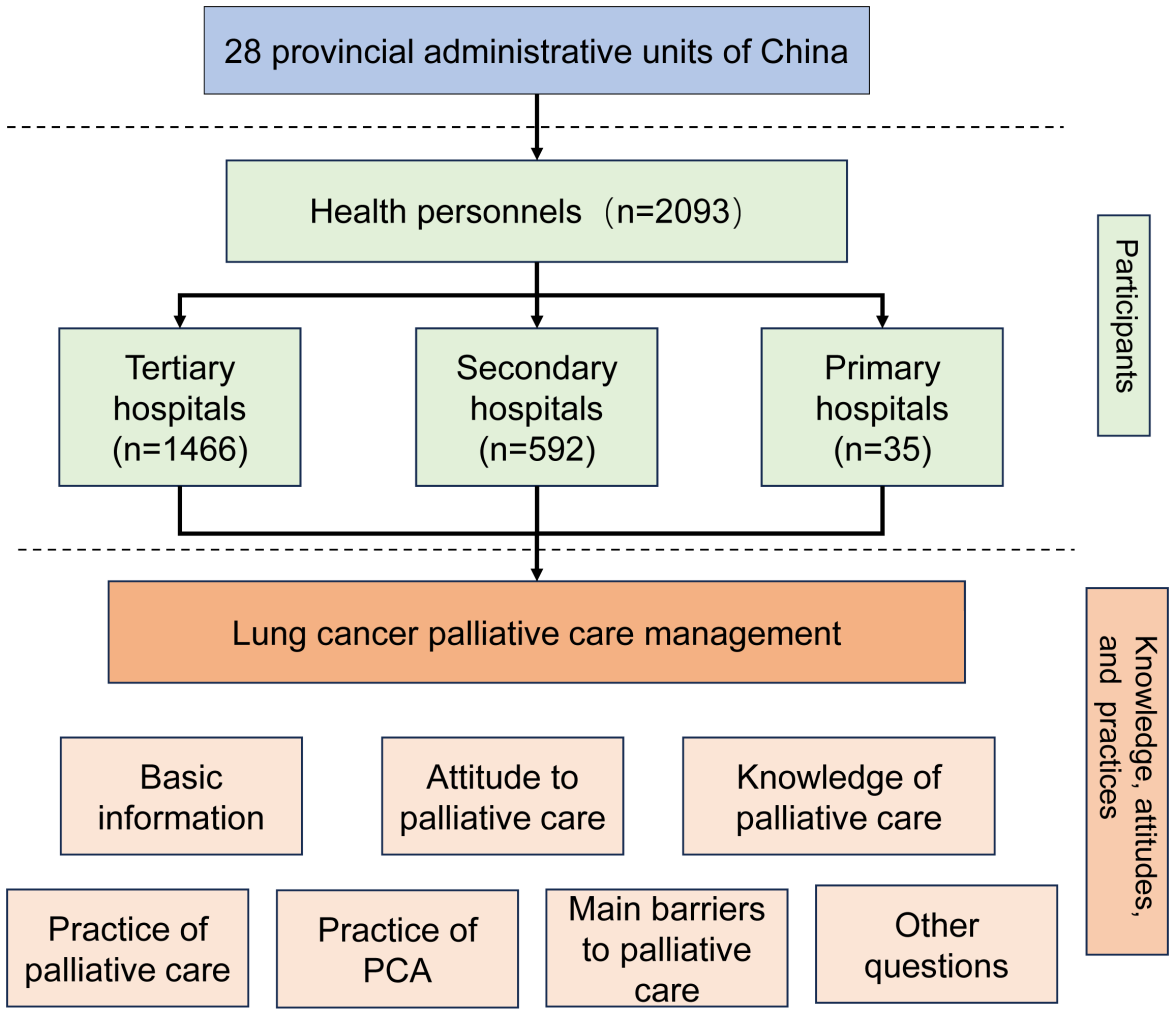
Framework of the study.

This nationwide, cross-sectional, descriptive study endeavored to elucidate the contemporary knowledge, attitudes, and practices pertinent to lung cancer palliative care (LCPC) management in China, as illustrated in **Figure 1**. This study was approved by the Ethics Committee of the Chongqing University Cancer Hospital (CZLS2022022-A).

The questionnaire was developed in strict accordance with the official consensus guidelines established by Chinese experts specializing in lung cancer palliative care, undergoing meticulous revision by an interdisciplinary expert panel composed of physicians, pharmacists, nurses, and epidemiologists from the Chongqing University Cancer Hospital. Prior to the commencement of the main survey, a rigorous quality control process was enforced throughout the design and administration phases of the questionnaires, which incorporated a pilot study with a limited sample size. Consequently, the validity and security of the questionnaire were thoroughly ensured.

From January 14, 2022, to March 1, 2022, 2093 participants from 28 provincial administrative units and 706 hospitals in China were enrolled among the associates of the China Anti-Cancer Association Cancer Rehabilitation and Palliative Care (CRPC) and Cancer Rehabilitation and Palliative Care Professional Committee of Chongqing Medical Biotechnology Association. Explicit informed consent was secured from every participant. The study was voluntary, and subjects were enrolled after a presentation of the research and possible conflicts of interest related to it. The participants were recruited from departments related to lung cancer palliative care (such as, Palliative care, Oncology, Respiratory, General, and other related departments), including physicians, nurses, and pharmacists, as the main body of the questionnaire survey of health personnels.

### Sample size calculating

A cross-sectional questionnaire survey was administered, employing a rigorous multi-stage random sampling methodology. Guided by the Kendall sample estimation method for multivariate analyses, it was established that the sample size should ideally span 5 to 10 times the count of variables under examination (17). Our survey encompassed a total of thirty questionnaire dimensions, thereby necessitating a minimum sample size of 150 to 300 participants. Ultimately, 2,093 individuals successfully completed the online questionnaire, fulfilling the essential criterion of an adequate sample size. Additionally, we utilized the G-power 3.1.9.7 software (Kiel University, Kiel, Germany) to execute a *post hoc* power analysis, evaluating the statistical power of our study. The computed power (1-β) was ascertained to be 0.997, considering an error probability (α or significance level) of 0.05 and a cumulative sample size of 2,093 participants.

### Survey questionnaire

The questionnaire comprised seven major components (30 questions), which are basic information (6 questions), attitude to palliative care (2 questions), knowledge related the palliative care symptom management (5 questions), the practice of palliative care management (5 questions), the practice of PCA (5 questions), main barriers to palliative care practices of lung cancer and PCA treatment (3 questions), and 4 other questions. In addition to basic information about the participants (sex, age, hospital grade, department, occupation, and professional title), a KAP assessment question about LCPC management was included. Online questionnaires were distributed via WeChat, and the respondent answered questionnaires via the Internet platform “Questionnaire Star” (https://www.wjx.cn/). The questionnaires were filled out by all participants voluntarily.

The questionnaire included three question types: single-choice, multiple-choice, and ranked. The participants had to complete the questionnaire based on the information obtained from their clinical practice over the past month. For ranking questions, the reverse scoring method was used to score and rank all options. For instance, if there were 8 options, the number of the first order was 1, the number of the eighth order was 8, the score of the first order was 8, the number of the second order was 7, and so on.

### Statistical analysis

SPSS 22.0 was used to analyze the data. The scores are expressed as mean ± standard deviations (SD). Demographics were reported with frequency and constituent ratio, and differences between groups were compared using the chi-square or Fisher exact probability test. The Kruskal-Wallis H test or Mann-Whitney U test was used to compare the distribution of ordered data between groups. Multiple choice questions used multiple response cross analysis and were described by response rate and percent of Cases (multiple responses). The percent of Cases (multiple responses) rate was used to analyze and compare the proportion of each selected option independently. The Spearman correlation coefficient (r) was used to assess the correlation. Statistical significance was defined as *P*< 0.05.

## Result

### Respondents’ characteristics

A total of 2093 questionnaires were disseminated, achieving a 100% return and validity rate. Participants hailed from 706 hospitals spread across 28 provincial jurisdictions within China. Refer to **Table 1** for a comprehensive overview of the demographic and professional attributes of the 2093 participants. The gender distribution revealed a near-equivalence, with 964 males and 1129 females participating. A significant majority of the respondents, accounting for 77.2%, were aged 40 years or younger. Approximately 70% of the participants were affiliated with Tertiary hospitals. Over half of the respondents (62.2%) were members of the oncology department. Among the participants, 66.2% were physicians, 24.2% were nurses, and 5.0% were pharmacists. The professional ranks of the respondents were distributed as follows: 22.8% held junior positions, 38.8% were intermediate-level professionals, and 38.4% were seniors.

**Table 1:** Characteristics of the participants (n = 2093).

Characteristic	Frequency (n)	Percentage (%)
C1: Sex		
Male	964	46.1
Female	1129	53.9
C2: Age (years)		
≤40	1615	77.2
>40	478	22.8
C3: Hospital grade		
Tertiary	1466	70.0
Secondary	592	28.3
Primary	35	1.7
C4: Department		
Palliative care	90	4.3
Oncology	1301	62.2
Respiratory	120	5.7
General	79	3.8
Other	503	24.0
C5: Occupation		
Physicians	1385	66.2
Pharmacists	104	5.0
Nurses	507	24.2
Other	97	4.6
C6: Professional title		
Junior	478	22.8
Intermediate	812	38.8
Senior	803	38.4

### Attitudes toward palliative care

Roughly 84.2% of the respondents affirmed the parity of importance between antitumor therapy and palliative care, as shown in **Supplementary Table S1**, and this perception did not vary significantly across hospital grade categories. As per the participant responses detailed in **Supplementary Table S2**, the foremost advantages of palliative care were identified as enhancements to patients’ quality of life, extending survival duration, and mitigating anxiety and depressive symptoms in patients.

### Current practices of lung cancer palliative care in China

Around 44.2% of the healthcare professionals were affiliated with institutions where less than one quarter of lung cancer patients accessed palliative care, contrasting with merely 17.5% who were associated with facilities where over half of such patients received it, as outlined in **Table 2**. Notably, there were considerable disparities in the timing of palliative care recommendation for cancer patients across different hospital grades, as demonstrated in **Table 2**. Regarding the query about the percentage of lung cancer patients under their care expressing high satisfaction with palliative care outcomes in the preceding month, the level of satisfaction was notably greater among those working in Tertiary hospitals as compared to those in Secondary and Primary hospitals (χ² = 27.402, *P*= 0.002).

**Table 2:** Participants’ current palliative care practices of lung cancer in China (n = 2093).

Item	Tertiary hospital	Secondary hospital	Primary hospital	Total	χ^2^	*P-Value*
**P1: The proportion of patients receiving palliative care in your setting in the past month**					10.489	0.106
≤25%	638(43.5)	270(45.6)	17(48.6)	925(44.2)		
26%~50%	586(39.9)	207(35.0)	10(28.6)	803(38.3)		
51%~75%	156(10.6)	85(14.4)	5(14.3)	246(11.7)		
≥76%	88(6.0)	30(5.1)	3(8.6)	121(5.8)		
**P2: When you recommend that patients start palliative care?**					28.18	**0.002**
Advanced tumor patients	366(24.9)	160(27.0)	8(22.9)	534(25.5)		
The estimated survival is less than 6 months	238(16.2)	90(15.2)	8(22.9)	336(16.0)		
Patients who are not eligible for anti-tumor therapy	239(16.3)	103(17.4)	1(2.9)	343(16.4)		
Patients with limited treatment costs.	33(2.2)	15(2.5)	3(8.6)	51(2.4)		
Patients present with symptoms requiring palliative care, regardless of the patient’s illness stage	581(39.6)	223(37.7)	13(37.1)	817(39.0)		
Other	11(0.7)	1(0.2)	2(5.7)	14(0.7)		
**P3: What percentage of lung cancer patients in your charge was very satisfied with the results of palliative care in the past month?**					27.402	**0.002**
≤10%	248(16.9)	134(22.6)	6(17.1)	388(18.5)		
11%~20%	247(16.8)	88(14.9)	2(5.7)	337(16.1)		
21%~40%	294(20.0)	126(21.3)	8(22.9)	428(20.4)		
41%~60%	306(20.8)	120(20.3)	9(25.7)	435(20.8)		
61%~80%	206(14.0)	89(15.0)	8(22.9)	303(14.5)		
≥81%	167(11.4)	35(5.9)	2(5.7)	204(9.7)		

Data are n (%). Percentages might not total 100% because of rounding.Shown the P values with statistical significance P < 0.05 in bold.

Pain, dyspnea, cough, anorexia and cachexia, nausea and vomiting, anxiety and depression, and fatigue emerged as the most prevalent symptoms necessitating palliative care among lung cancer patients. Concurrently, as evidenced in **Supplementary Table S3**, pain, dyspnea, and cough were the top three concerns that both patients and their family members sought relief from.

### Knowledge related to the palliative care symptom management of lung cancer

**Supplementary Table S4** documents the participants’ responses concerning their familiarity with managing symptoms related to lung cancer palliative care. Strikingly, 70.6% of the participants provided incorrect answers when queried about managing cough in lung cancer patients under palliative care, reflecting a notable knowledge gap in this crucial area. On the other hand, around half of the participants managed to answer questions pertaining to nutrition accurately in the context of lung cancer palliative care, suggesting that this domain was somewhat better understood among the surveyed healthcare professionals.

### Barriers toward to palliative care practices of lung cancer

**Table 3** illustrates the prevailing concerns of the respondents regarding the challenges faced in implementing palliative care practices for lung cancer patients. Among the top-ranked impediments highlighted by the respondents were the concern among patients and their families about the safety of long-term usage of palliative medications, the misconception among patients and their families that palliative care is solely required during the terminal phase, and the tendency of medical personnel to prioritize anti-tumor treatments over palliative care initiatives. Additionally, **Table 3** reveals insights into how respondents perceive the role of palliative care medications. With respect to these issues, there exist significant discrepancies in the opinions of medical staff across various hospital tiers, as evidenced by statistically significant chi-squared values (χ2 = 431.156, *P* < 0.001 for question B1, and χ2 = 150.485, *P* < 0.001 for question B2).

**Table 3:** Main barriers to participants’ palliative care practices of lung cancer in China (n=2093).

Item	Tertiary hospital(n=1466)			Secondary hospital(n=592)			Primary hospital(n=35)			Total
B1: The main barriers to palliative care affecting lung cancer patients	n	Response (%)	Percent of Cases (Multiple Response)(%)	n	Response (%)	Percent of Cases (Multiple Response)(%)	n	Response (%)	Percent of Cases (Multiple Response)(%)	
Patients and families are concerned about the safety of long-term use of palliative care-related drugs	1180	16.5	80.5	500	16.6	84.5	23	17.4	65.7	1703
Patients and their families have a wrong perception of palliative care, believing that only end-stage patients need palliative care	1103	15.4	75.2	467	15.5	78.9	21	15.9	60.0	1591
Medical staff pay less attention to palliative care and pay more attention to anti-tumor treatment	976	13.7	66.6	423	14.0	71.5	21	15.9	60.0	1420
Palliative care is considered by medical staff to increase the workload	986	13.8	67.3	408	13.5	68.9	17	12.9	48.6	1411
Medical staff do not know the expertise of palliative care	781	10.9	53.3	315	10.4	53.2	13	9.8	37.1	1109
Patients and their families consider palliative care too expensive	739	10.3	50.4	292	9.7	49.3	15	11.4	42.9	1046
Unsatisfied with the efficacy of symptom management medications	728	10.2	49.7	323	10.7	54.6	11	8.3	31.4	1062
Unsatisfied with adverse reactions to symptom management medications	649	9.1	44.3	287	9.5	48.5	11	8.3	31.4	947
Total	7142	100.0	487.2	3015	100	509.3	132	100	377.1	10289
B2: The purpose of palliative care medication										
The rational use of palliative care drugs can effectively reduce the physical and mental pain of patients and improve their quality of life	1270	27.4	86.6	512	27.1	86.5	27	27.3	77.1	1809
Patients receiving palliative care have a long pre-survival period. In clinical medication, intervention and improvement should be carried out to remove the uncomfortable symptoms generated in the course of disease treatment to improve the quality of life of patients, and appropriate extension of the survival time of patients should also be considered	1249	26.9	85.2	521	27.6	88.0	28	28.3	80.0	1798
Rational use of palliative care drugs to improve physical and/or psychosomatic symptoms in end-stage patients	1265	27.3	86.3	503	26.6	85.0	26	26.3	74.3	1794
The principle of palliative medicine is that relief of symptoms takes precedence over delayed survival	853	18.4	58.2	353	18.7	59.6	18	18.2	51.4	1224
**Total**	4637	100	316.3	1889	100	319.1	99	100.1	282.9	6625

χ2 = 431.156, P < 0.001 for question B1, and χ2 = 150.485, P < 0.001 for question B2.

Data are n (%). Percentages might not total 100% because of rounding.

### Practices toward cancer pain management of lung cancer in China

**Table 4** provides an overview of the prevailing strategies for managing cancer pain among Chinese healthcare providers. About 58.4% of the respondents indicated that ≤ 20% of their refractory cancer pain patients experienced inadequate pain control or unbearable side effects. Patient-Controlled Analgesia (PCA) delivers outstanding analgesic benefits for patients experiencing refractory cancer pain (18). The leading three rationales for employing PCA therapy included patients who require high-dose opioids systemically or exhibit intolerance to opioid side effects (31.1%), cancer patients with swallowing difficulties or gastrointestinal impairments (21.8%), and addressing recurrent episodes of pain (21.3%). When administering analgesics via PCA for cancer pain, the majority (76.9%) of healthcare providers opted for strong opioid μ-agonists like hydromorphone, morphine, and sufentanil. Statistically significant differences (*P* < 0.05) were observed in responses to these questions based on the hospital grade category. Among the prevalent treatment approaches for patients suffering from refractory cancer pain were PCA (32.8%), high-dose oral opioids (28.9%), and a combination of systemic and local therapies (24.2%).

**Table 4:** Participants’ current pain management practices of lung cancer in China (n = 2093).

Item	Tertiary hospital	Secondary hospital	Primary hospital	Total	χ^2^	*P-value*
**P4: The approximate percentage of patients who receive 300 mg/d (equivalent oral morphine dose) or more of strong opioids for analgesia**					17.767	**0.007**
≤20%	714(48.6)	316(53.4)	9(25.7)	1039(49.6)		
21%~40%	533(36.3)	196(33.1)	14(40.0)	743(35.5)		
41%~60%	166(11.3)	64(10.8)	9(25.7)	239(11.4)		
≥61%	55(3.7)	16(2.7)	3(8.6)	74(3.5)		
**P5: The approximate percentage of patients who receive a strong opioid analgesic orally at or above 300 mg/d (equivalent oral morphine dose) but whose pain is poorly controlled or whose adverse reactions are not tolerated**					21.982	**0.001**
≤20%	832(56.7)	379(64.0)	12(34.3)	1223(58.4)		
21%~40%	438(29.8)	147(24.8)	13(37.1)	598(28.5)		
41%~60%	160(10.9)	58(9.8)	8(22.9)	226(10.8)		
≥61%	38(2.6)	8(1.4)	2(5.7)	48(2.3)		
**P6: The most common reason for PCA treatment**					25.326	**0.005**
Patients with systemic application of large doses of opioids or adverse reactions to opioids that cannot be tolerated	487(33.2)	154(26.0)	11(31.4)	652(31.1)		
Cancer pain patients with dysphagia or gastrointestinal dysfunction	298(20.3)	146(24.7)	12(34.3)	456(21.8)		
Management of frequent outbreaks of pain	297(20.2)	146(24.7)	4(11.4)	447(21.3)		
Dose titration of opioids	204(13.9)	78(13.2)	3(8.6)	285(13.6)		
Analgesic treatment for patients with terminal cancer pain	162(11.0)	62(10.5)	3(8.6)	227(10.8)		
Other	20(1.4)	6(1.0)	2(5.7)	28(1.3)		
**P7: The most commonly used analgesic drugs for PCA treatment of cancer pain**					30.434	**0.001**
Strong opioid μ-agonists, such as hydromorphone, morphine, and sufentanil	1119(76.2)	473(79.9)	20(57.1)	1612(76.9)		
μ-receptor partial agonist (e.g., buprenorphine	74(5.0)	17(2.9)	2(5.7)	93(4.4)		
μ-receptor excitation-antagonist (butorphanol, desoxine, pentazoxin, nalbuphine)	188(12.8)	60(10.1)	7(20.0)	255(12.2)		
Pethidine	31(2.1)	13(2.2)	4(11.4)	48(2.3)		
Tramadol	48(3.3)	27(4.6)	1(2.9)	76(3.6)		
NSAID drugs	8(0.5)	2(0.3)	1(2.9)	11(0.5)		
**P8: The treatment of refractory cancer pain is often used**					14.025	0.081
High dose oral opioid	403(27.5)	192(32.4)	10(28.6)	605(28.9)		
Patch	174(11.9)	87(14.7)	7(20.0)	268(12.8)		
PCA	500(34.1)	179(30.2)	9(25.7)	688(32.8)		
Systemic administration combined with local treatment	370(25.2)	129(21.8)	8(22.9)	507(24.2)		
Other	21(1.4)	5(0.8)	1(2.9)	27(1.3)		

Data are n (%). Percentages might not total 100% because of rounding.Shown the P values with statistical significance P < 0.05 in bold.

### Barriers toward PCA treatment of cancer pain

The distribution of barriers to PCA treatment for cancer pain among medical staff across hospital grade levels exhibits significant variability, as evidenced by the goodness of fit test (χ2 = 620.022, *P* < 0.001, **Table 5**). In Secondary and Tertiary hospitals, the three most frequently identified and ranked concerns were: (1) fear of adverse drug reactions from overdosing, (2) apprehension about opioid addiction, and (3) the increased financial burden on patients due to PCA treatment.

**Table 5:** Main barriers to different hospital grades in PCA treatment of cancer pain (n = 2093).

Item	Tertiary hospital(n=1466)			Secondary hospital(n=592)			Primary hospital(n=35)			Total
B3: Barriers to PCA treatment of cancer pain	n	Response (%)	Percent of Cases (Multiple Response)(%)	n	Response (%)	Percent of Cases (Multiple Response)(%)	n	Response(%)	Percent of Cases (Multiple Response)(%)	
Worried about adverse reactions to drug overdose	834	21.7	56.9	351	21.1	59.3	16	18.6	45.7	1201
Worried about opioid addiction	815	21.2	55.6	340	20.5	57.4	21	24.4	60.0	1176
Increase the financial burden of patients	791	20.6	54.0	320	19.3	54.1	20	23.3	57.1	1131
The popularity is not enough, many hospitals do not have PCA pumps	477	12.4	32.5	198	11.9	33.4	10	11.6	28.6	685
Equipment or operation technical obstacles	687	17.9	46.9	342	20.6	57.8	14	16.3	40.0	1043
It is not consistent with our related administration policy of hemp and essence drugs	241	6.3	16.4	109	6.6	18.4	5	5.8	14.3	355
**Total**	3845	100.1	262.3	1660	100	280.4	86	100	245.7	5591

χ^2^ = 620.022, P<0.001.

Data are n (%). Percentages might not total 100% because of rounding.

In contrast, at the Primary healthcare level, the barriers most often encountered comprised: (i) the fear of opioid dependency, (ii) the exacerbation of patient economic hardship due to treatment costs, and (iii) similar concerns over the potential adverse effects of drug overdoses. These findings collectively illuminate the divergent spectra of challenges influencing PCA treatment acceptance across different tiers of the healthcare system.

### Correlations between participants’ characteristics and palliative care practices of lung cancer

In this study, a significant inverse correlation was observed between the frequency of “patients receiving palliative care” and the department hierarchy (Pearson’s correlation coefficient r_s_ = -0.145, *P* < 0.01). Additionally, the degree of satisfaction among lung cancer patients (“percentage of patients reporting very high satisfaction”) with the outcomes of palliative care showed a negative association with hospital grading, becoming less satisfactory as the hospital level decreased (r_s_ = -0.057, *P* < 0.01).

Furthermore, the phenomenon of “poorly controlled pain or intolerable adverse reactions” in patients was negatively correlated with participants’ age (r_s_ = -0.058, *P* < 0.01), suggesting that younger health personnels were more likely to experience inadequate pain management or adverse effects. Conversely, this issue displayed a positive correlation with the professional background of the respondents (r_s_ = 0.105, P-value < 0.01), implying that it was more prevalent among certain occupational groups. All these correlations are detailed in **Supplementary Table S5**.

### Cognition of participants in different grades of hospitals on issues related to the establishment of expert consensus in palliative care

**Supplementary Table S6** discloses statistically significant variances in the distribution of responses across different hospital grades for two inquiries: the presence of a “specific pathway or standardized treatment procedure for cancer palliative care in their department” (χ² = 33.431, *P* < 0.001), and whether participants “had engaged in online or offline training related to lung cancer palliative care (LCPC) within the last two years” (χ² = 16.123, *P* = 0.013).

According to the table, 43.2% of all participants did not have a designated palliative care pathway or standardized treatment protocol for cancer patients. Concurrently, 18.7% of the medical staff members were part of departments that nominally possessed a standard palliative care pathway but failed to implement it rigorously.

An overwhelming majority (97.5%) of the respondents considered it imperative to develop a clear expert consensus on palliative care, aiming to standardize and streamline processes ranging from symptom screening and assessment to treatment, nursing, and home management for lung cancer patients. Within the suggested consensus framework, the top three priorities highlighted were: the establishment of a practical diagnosis and treatment process/pathway, further elucidation of the conceptual understanding and value of palliative care, and provision of treatment guidelines and medication recommendations tailored to different symptom presentations.

Regarding the participation in LCPC training activities over the previous 24 months, a notable 33.9% of the participants had not taken part in either online or offline educational sessions.

## Discussion

Our KAP study represents a pioneering endeavor in conducting a comprehensive, large-scale exploration of Lung Cancer Palliative Care management in China and inaugurates an in-depth examination of PCA for refractory cancer pain management. In the ethical pursuit of expanding palliative care access in LMICs, understanding and addressing health personnel’s baseline knowledge and attitudes through training is crucial. Most participants (84.2%) agreed that anti-tumor therapy and palliative care are equally important, and 39% acknowledged the need for palliative care regardless of illness stage once symptoms warrant it. Despite this recognition, 82.5% of lung cancer patients didn’t receive palliative care, and only 24.2% expressed high satisfaction levels.

There were marked discrepancies in palliative care understanding and practices across different hospital grades, particularly in Secondary and Primary hospitals needing immediate improvement. Alarmingly, 33.9% hadn’t undergone LCPC-related training in the last 24 months, underscoring a pressing need for education. Key symptoms demanding palliative care in lung cancer patients include pain, dyspnea, cough, anorexia and cachexia, nausea, and vomiting; however, misconceptions and fears around opioid adverse reactions persist worldwide (13, 18, 19), although Vietnam has seen lasting improvements following formal courses (15).

While Chinese health personnel increasingly recognize cancer pain, challenges remain in effective management. International research confirms that a lack of professional skills and training hinders optimal pain control (19–21). Our study revealed similar barriers to those reported globally, with concerns over long-term drug safety and misconceptions that palliative care is reserved for end-stage patients (22–27). These findings underscore the urgency to educate health personnel in lung cancer pain management and emphasize the need for enhanced cancer pain management generally, along with patient education on opioid use.

PCA offers a more efficient analgesic approach that improves pain relief and patient satisfaction (28, 29). About 32.8% of participants viewed PCA as suitable for refractory cancer pain cases, primarily due to patients experiencing systemic high-dose opioid use or intolerable side effects. Tertiary hospitals predominantly used PCA (34.1%), while Secondary and Primary hospitals relied on high-dose oral opioids, possibly reflecting insufficient PCA-specific training. To standardize treatment for intractable pain, health personnel require accessible PCA training and implementation.

Among barriers to PCA adoption for cancer pain management, Chinese participants cited concerns about overdose adverse reactions, opioid addiction, and increased patient financial burden (18). While acknowledging historical and social reasons behind opiophobia, physicians must confidently prescribe morphine according to national guidelines without fearing overdose risks to provide high-quality palliative care.

The ongoing public health discourse underscores how social determinants influence cancer screening behavior and can mitigate disparities by promoting early detection (30). With a focus on LCPC management, our study emphasizes the importance of contextualizing findings within the broader social determinants framework to address screening inequalities. Barriers like limited rural healthcare access, low health literacy, financial constraints, and inadequate insurance often impede early detection initiatives (30, 31).

Addressing these underlying factors can lighten the load on palliative care by encouraging early detection and proactive disease management. Thus, we advocate for multidimensional, interdisciplinary strategies targeting relevant social determinants to enhance lung cancer palliative care management and, subsequently, cancer screening equity. This could encompass community health education, expanded coverage policies, cost reduction measures, targeted outreach, and primary care partnerships to streamline referrals.

### Limitations

Despite rigorous survey design, execution, and analysis, the research team acknowledges several limitations. Firstly, the scarcity of prior research on LCPC in China means our findings await external validation across diverse settings. Secondly, restricting the sample to health professionals from CRPC Special Committee member units potentially limits the generalizability of results to broader healthcare contexts in China. Thirdly, the reliance on self-reported data may introduce recall bias, potentially distorting participants’ reports of experiences and practices. Lastly, while the sample size is substantial, it is relatively small compared to the multitude of healthcare facilities across China, meaning temporal and geographical variations in clinical practice could impact observed associations without being fully captured in our data. Hence, future studies employing larger, more representative samples and robust validation methods are needed to strengthen the validity and applicability of these findings.

### Recommendations for future research

Firstly, to validate and generalize our survey’s conclusions, it’s essential to replicate the study across a wider range of geographical locations and healthcare settings, encompassing urban and rural areas, various hospital tiers, and community clinics. Such diversity would yield a more accurate representation of the national scenario and greater generalizability.

Secondly, longitudinal studies could monitor changes in health personnel’s KAP and assess the effectiveness of new policies, training programs, or interventions. This kind of research would serve as a monitoring tool guiding future improvements in LCPC.

Thirdly, prioritizing targeted, intervention-oriented research includes developing and evaluating innovative educational programs, mentorship structures, or quality improvement projects to fill knowledge gaps and shift negative attitudes.

Moreover, leveraging technological advancements is increasingly important. Future research should examine the role of digital health tools, including telepalliative care and e-learning platforms, in enhancing health personnel’s knowledge and practice in LCPC.

In conclusion, exploring these multifaceted research avenues significantly contributes to refining and elevating the standards of lung cancer palliative care in China, ensuring patients receive timely, effective, and compassionate end-of-life care. Adhering to these recommendations, future research will indubitably play a pivotal role in advancing LCPC management in China.

### Clinical implications for health managers and policymakers

The clinical implications derived from KAP research in LCPC management provide actionable insights for health managers and policymakers. Health managers should use these insights to establish or fortify education and training programs to bridge identified knowledge gaps and reshape attitudes. They can also apply these findings to create performance improvement plans and redesign workforce strategies, perhaps by increasing staffing or redistributing responsibilities to optimize LCPC services.

Policymakers can use the research to guide legislative and regulatory reforms conducive to a more supportive environment for LCPC integration within healthcare systems. This might involve tackling structural barriers, allocating funds to strengthen infrastructure, staffing, and access to services, particularly in underprivileged areas. Furthermore, this research informs the development of national strategies for cancer control and palliative care, aligning public health policies with contemporary best practices and responding to the unique needs uncovered by the study.

In summary, the clinical implications emerging from KAP research in LCPC management provide a strategic blueprint for health managers and policymakers to implement evidence-based changes that enhance the quality of care and meet the intricate needs of lung cancer patients and their families within the healthcare system.

## Conclusion

The present examination of Chinese healthcare professionals has exposed a profound requirement for targeted training in LCPC, with particular emphasis on PCA in managing refractory cancer pain. Additionally, there exists an imperative need to forge a lucid expert consensus on palliative care, with the aim of standardizing the processes of screening, evaluation, treatment, nursing care, and home-based symptom management for lung cancer patients. Healthcare professionals must undergo rigorous training and be adequately equipped to deliver high-quality palliative care, a prerequisite for ensuring universal access to these vital services.

## Data availability statement

The raw data supporting the conclusions of this article will be made available by the authors, without undue reservation.

## Ethics statement

This study was approved by the Ethics Committee of Chongqing University Cancer Hospital (CZLS2022022-A). The studies were conducted in accordance with the local legislation and institutional requirements. The participants provided their written informed consent to participate in this study.

## Author contributions

MC: Data curation, Formal analysis, Resources, Supervision, Writing – original draft, Writing – review & editing. SH: Data curation, Formal analysis, Writing – review & editing. HQY: Project administration, Writing – review & editing. YH: Data curation, Formal analysis, Investigation, Writing – review & editing. HY: Data curation, Investigation, Methodology, Writing – review & editing. LY: Investigation, Writing – review & editing. LT: Investigation, Writing – review & editing. SW: Investigation, Writing – review & editing.
