# Erratum: Knowledge, attitudes, and current practices toward lung cancer palliative care management in China: a national survey

**DOI:** 10.3389/fonc.2024.1516468

**Published:** 2024-11-05

**Authors:** Frontiers Production Office

**Affiliations:** Frontiers Media SA, Lausanne, Switzerland

**Keywords:** palliative care, China, surveys and questionnaires, pain management, health personnel

Due to a production error, there was an error in the footnote in the list of authors. Instead of “**^†^** These authors have contributed equally to this work” being applied to the second and the third authors, the statement should read “**^†^** These authors have contributed equally to this work and share first authorship” and should be applied to the first three authors. The publisher apologizes for this mistake.

The original version of this article has been updated.

