# Supplementary material for: Knowledge, attitudes, and current practices toward lung cancer palliative care management in China: a national survey
Source: Front Oncol. 2024 May 15;14:1382496. doi: 10.3389/fonc.2024.1382496 (PMC11133550; doi:10.3389/fonc.2024.1382496)
Supplement: Supplementary file 1 [file DataSheet_1.doc]

**Supplementary Table S1. Participants’** current palliative care attitudes of lung cancer in China (n = 2093).

| **Item** | **Tertiary**  **hospital** | **Secondary hospital** | **Primary**  **hospital** | **Total** | **χ^2^** | ***P-Value*** |
| --- | --- | --- | --- | --- | --- | --- |
| **A1: The importance of palliative and** **Anti-tumor therapy for cancer patients** |  |  |  |  | 8.481 | 0.205 |
| Anti-tumor therapy is more important | 207（13.3） | 43（10.1） | 9（8.0） | 259（12.4） |  |  |
| Palliative care is more important | 48（3.1） | 9（2.1） | 5（4.4） | 62（3.0） |  |  |
| Equally important | 1297（83.2） | 369（87.0） | 99（87.6） | 1765（84.2） |  |  |
| Neither | 6（0.4） | 3（0.7） | 0（0） | 9（0.4） |  |  |

Data are n (%). Percentages might not total 100% because of rounding.
