# Supplementary material for: Knowledge, attitudes, and current practices toward lung cancer palliative care management in China: a national survey
Source: Front Oncol. 2024 May 15;14:1382496. doi: 10.3389/fonc.2024.1382496 (PMC11133550; doi:10.3389/fonc.2024.1382496)
Supplement: Supplementary file 2 [file DataSheet_2.doc]

**Supplementary Table S2. The most essential benefits that palliative care can bring.**

| **Item** | **Score** |
| --- | --- |
| A2: Which one is the most important benefits that palliative care can bring? | |
| Improve patients' quality of life | 5.21±1.69 |
| Prolong the survival of patients | 4.00±1.99 |
| Improve anxiety and depression in patients | 3.52±1.63 |
| Reduce depression and improve the quality of life of caregivers | 2.26±1.58 |
| Save the total cost of patient treatment | 2.09±1.54 |
| Improve the treatment satisfaction of patients and their families and improve the doctor-patient relationship | 1.56±1.35 |

Data are means ±SD.
