# Supplementary material for: Knowledge, attitudes, and current practices toward lung cancer palliative care management in China: a national survey
Source: Front Oncol. 2024 May 15;14:1382496. doi: 10.3389/fonc.2024.1382496 (PMC11133550; doi:10.3389/fonc.2024.1382496)
Supplement: Supplementary file 3 [file DataSheet_3.doc]

**Supplementary Table S3.** Symptoms in lung cancer patients that require palliative care.

| **Item** | **Score** |
| --- | --- |
| Please rank the following common symptoms in lung cancer patients that require palliative care, starting with the symptoms you think are the most common（Ranking question） | |
| Pain | 6.00±2.54 |
| Dyspnea | 4.99±2.72 |
| Cough | 4.99±3.07 |
| Anorexia and cachexy | 4.47±2.42 |
| Nausea and Vomiting | 3.83±2.30 |
| Fatigue | 2.81±2.12 |
| Anxiety and Depression | 2.74±2.21 |
| Other | 0.36±0.91 |
| If you are a patient or a family member of a patient, what are the symptoms that affect your quality of life the most and would like to be resolved? Start with the symptoms that you think have the most impact（Ranking question） | |
| Pain | 6.44±2.44 |
| Dyspnea | 5.45±2.68 |
| Cough | 4.49±2.92 |
| Anorexia and cachexy | 4.39±2.38 |
| Nausea and Vomiting | 4.01±2.26 |
| Anxiety and Depression | 2.67±2.11 |
| Fatigue | 2.54±1.85 |
| Other | 0.29±0.74 |

Data are means ± SD.
