# Supplementary material for: Knowledge, attitudes, and current practices toward lung cancer palliative care management in China: a national survey
Source: Front Oncol. 2024 May 15;14:1382496. doi: 10.3389/fonc.2024.1382496 (PMC11133550; doi:10.3389/fonc.2024.1382496)
Supplement: Supplementary file 4 [file DataSheet_4.doc]

**Supplementary Table S4. Participants’ knowledge related to the palliative care symptom management of lung cancer in China (n=2093).**

| **Item** | **Tertiary**  **hospital** | **Secondary hospital** | **Primary**  **hospital** | **Total** | **χ^2^** | ***P-Value*** |
| --- | --- | --- | --- | --- | --- | --- |
| **K1: Lung cancer cough** |  |  |  |  | 0.732 | 0.693 |
| correct | 432（29.5） | 175（29.6） | 8（22.9） | 615（29.4） |  |  |
| Not Correct | 1034（70.5） | 417（70.4） | 27（77.1） | 1478（70.6） |  |  |
| **K2: Nutritional screening and evaluation** |  |  |  |  | 0.852 | 0.653 |
| correct | 665（45.4） | 261（44.1） | 18（51.4） | 944（45.1） |  |  |
| Not Correct | 801（54.6） | 331（55.9） | 17（48.6） | 1149（54.9） |  |  |
| **K3: Anorexia and cachexy** |  |  |  |  | 10.991 | **0.004** |
| correct | 809（55.2） | 307（51.9） | 10（28.6） | 1126（53.8） |  |  |
| Not Correct | 657（44.8） | 285（48.1） | 25（71.4） | 967（46.2） |  |  |
| **K4: Parenteral nutrition** |  |  |  |  | 11.812 | **0.003** |
| correct | 909（62.0） | 407（68.8） | 17（48.6） | 1333（63.7） |  |  |
| Not Correct | 557（38.0） | 185（31.3） | 18（51.4） | 760（36.3） |  |  |
| **K5: Parenteral nutrition nursing support** |  |  |  |  | 8.760 | **0.013** |
| correct | 762（52.0） | 340（57.4） | 13（37.1） | 1115（53.3） |  |  |
| Not Correct | 704（48.0） | 252（42.6） | 22（62.9） | 978（46.7） |  |  |

Data are n (%). Percentages might not total 100% because of rounding.
