# Supplementary material for: Knowledge, attitudes, and current practices toward lung cancer palliative care management in China: a national survey
Source: Front Oncol. 2024 May 15;14:1382496. doi: 10.3389/fonc.2024.1382496 (PMC11133550; doi:10.3389/fonc.2024.1382496)
Supplement: Supplementary file 5 [file DataSheet_5.doc]

**Supplementary Table S5. Correlations between participants’ characteristics and palliative care Practices of lung cancer in China (r_s_, n = 2093).**

| **Variable** | Sex | Age | Hospital grade | Department | Occupation | Professional title |
| --- | --- | --- | --- | --- | --- | --- |
| P1:The proportion of patients receiving palliative care in your setting in the past month | 0.055* | -0.026 | -0.004 | -0.145** | 0.036 | -0.013 |
| P3:What percentage of lung cancer patients in your charge were very satisfied with the results of palliative care in the past month? | 0.006 | -0.039 | -0.057** | -0.069** | -0.001 | -0.037 |
| P5:The approximate percentage of patients who receive a strong opioid analgesic orally at or above 300mg/d(equivalent oral morphine dose), but whose pain is poorly controlled or whose adverse reactions are not tolerated | 0.027 | -0.058** | -0.044* | -0.025 | 0.105** | -0.056* |

Note: **P* ˂ 0.05, ***P* ˂ 0.01.
