# Supplementary material for: Knowledge, attitudes, and current practices toward lung cancer palliative care management in China: a national survey
Source: Front Oncol. 2024 May 15;14:1382496. doi: 10.3389/fonc.2024.1382496 (PMC11133550; doi:10.3389/fonc.2024.1382496)
Supplement: Supplementary file 6 [file DataSheet_6.doc]

**Supplementary Table S6. Cognition of participants in different hospital grades on issues related to the establishment of expert consensus in palliative care (n = 2093).**

| **Project** | **Tertiary**  **hospital** | **Secondary hospital** | **Primary**  **hospital** | **Total** | | **χ^2^** | ***P*-Value** |
| --- | --- | --- | --- | --- | --- | --- | --- |
| **Is there a specific pathway or standardized treatment procedure for cancer palliative care in your department?** |  |  |  |  | | 33.431 | **＜0.001** |
| No | 594（40.5） | 290（49.0） | 21（60.0） | 905（43.2） | |  |  |
| Yes, and strictly enforced | 615（41.9） | 173（29.2） | 11（31.4） | 799（38.1） | |  |  |
| Yes, but it's not strictly enforced | 259（17.6） | 129（21.8） | 3（8.6） | 391（18.7） | |  |  |
| **Whether there is a need for a clear expert consensus on palliative care to regulate issues such as screening, assessment, treatment, care and home-based management of lung cancer patients with related symptoms** |  |  |  |  | | 0.884 | 0.643 |
| Yes, it is necessary. | 1432（97.5） | 578（97.6） | 35（0） | 2057（97.5） | |  |  |
| No, there is no need. | 36（2.5） | 14（2.4） | 0（0） | 36（2.5） | |  |  |
| **If there is an expert consensus on palliative care for lung cancer, what guidance would you most like to focus on in the consensus** |  |  |  |  | | 10.871 | 0.368 |
| Further clarify the concept and value of palliative care | 383（26.1） | 161（27.2） | 10（28.6） | 554（26.4） |  | |  |
| Practical diagnosis and treatment procedures and pathways | 436（29.7） | 166（28.0） | 6（17.1） | 608（29.0） |  | |  |
| Different symptom assessment principles and related assessment scales | 199（13.6） | 65（11.0） | 5（14.3） | 269（12.8） |  | |  |
| Treatment specifications and medication guidelines for different symptoms | 242（16.5） | 112（18.9） | 6（17.1） | 360（17.2） |  | |  |
| Nursing norms and operational procedures for different symptoms | 130（8.9） | 64（10.8） | 5（14.3） | 199（9.5） |  | |  |
| **Have you participated in online or offline training related to lung cancer palliative care in the past 24 months** |  |  |  |  | 16.123 | | **0.013** |
| Participated in both online and offline training | 532（36.2） | 181（30.6） | 12（34.3） | 725（34.6） |  | |  |
| Only participated in online training | 362（24.7） | 137（23.1） | 5（14.3） | 504（24.1） |  | |  |
| Only participated in offline training | 114（7.8） | 39（6.6） | 3（8.6） | 156（7.4） |  | |  |
| None | 460（31.3） | 235（39.7） | 15（42.9） | 710（33.9） |  | |  |

Data are n (%). Percentages might not total 100% because of rounding.
